# Multilocus Sequence Typing Breathes Life into a Microbial Metagenome

**DOI:** 10.1371/journal.pone.0000017

**Published:** 2006-12-20

**Authors:** Eshwar Mahenthiralingam, Adam Baldwin, Pavel Drevinek, Elke Vanlaere, Peter Vandamme, John J. LiPuma, Chris G. Dowson

**Affiliations:** 1 Cardiff School of Biosciences, Cardiff University, Cardiff, United Kingdom; 2 Department of Biological Sciences, Warwick University, Coventry, United Kingdom; 3 Laboratorium voor Microbiologie, Universiteit Gent, Gent, Belgium; 4 Department of Pediatrics, University of Michigan, Ann Arbor, Michigan, United States of America; University of Maryland, United States of America

## Abstract

Shot-gun sequencing of DNA isolated from the environment and the assembly of metagenomes from the resulting data has considerably advanced the study of microbial diversity. However, the subsequent matching of these hypothetical metagenomes to cultivable microorganisms is a limitation of such cultivation-independent methods of population analysis. Using a nucleotide sequence-based genetic typing method, multilocus sequence typing, we were able for the first time to match clonal cultivable isolates to a published and controversial bacterial metagenome, *Burkholderia* SAR-1, which derived from analysis of the Sargasso Sea. The matching cultivable isolates were all associated with infection and geographically widely distributed; taxonomic analysis demonstrated they were members of *Burkholderia cepacia* complex Group K. Comparison of the *Burkholderia* SAR-1 metagenome to closely related *B. cepacia* complex genomes indicated that it was greater than 98% intact in terms of conserved genes, and it also shared complete sequence identity with the cultivable isolates at random loci beyond the genes sampled by the multilocus sequence typing. Two features of the extant cultivable clones support the argument that the *Burkholderia* SAR-1 sequence may have been a contaminant in the original metagenomic survey: (i) their growth in conditions reflective of sea water was poor, suggesting the ocean was not their preferred habitat, and (ii) several of the matching isolates were epidemiologically linked to outbreaks of infection that resulted from contaminated medical devices or products, indicating an adaptive fitness of this bacterial strain towards contamination-associated environments. The ability to match identical cultivable strains of bacteria to a hypothetical metagenome is a unique feature of nucleotide sequence-based microbial typing methods; such matching would not have been possible with more traditional methods of genetic typing, such as those based on pattern matching of genomic restriction fragments or amplified DNA fragments. Overall, we have taken the first steps in moving the status of the *Burkholderia* SAR-1 metagenome from a hypothetical entity towards the basis for life of cultivable strains that may now be analysed in conjunction with the assembled metagenomic sequence data by the wider scientific community.

## INTRODUCTION

The ability to assemble complete genomes from DNA extracted from the environment has revolutionized the study of uncultivated microorganisms and established the science of metagenomics [1]. Such cultivation-independent methods based on sequence analysis of DNA have considerably enhanced our understanding of the organism and functional diversity that occurs in many ecologically important environments, with marine microbial communities being one of the most studied using metagenomic methods [2]. Venter et al. [3] published one of the largest metagenomic datasets in 2004, comprising 1 billion bases of nucleotide sequence and defining 1800 genomic species in microbial populations within the Sargasso Sea. Several complete plasmids, bacteriophage and microbial genomes were identified in this sequence dataset. However, one of the bacterial metagenomes defined, *Burkholderia* SAR-1 [3], raised several concerns about the validity of the original metagenomic survey [2]: (i) the majority of sequencing fragments that formed the *Burkholderia* SAR-1 metagenome derived from just one of the sample sites in the Sargasso survey [3]; (ii) the metagenomic assembly demonstrated an absence of single nucleotide polymorphisms normally associated with DNA sequences from closely related microbial populations that continually mix within the open ocean, and finally (iii) *Burkolderia* species bacteria are typically considered terrestrial rather than oceanic. These features of the *Burkholderia* SAR-1 metagenome suggested that it may have derived from sample contamination [2] and illustrate that great care must be taken in sampling DNA to ensure that the organisms observed in metagenomic datasets are truly representative of the environment being studied [1]. Despite this controversy, the Sargasso Sea dataset represented a ground-breaking metagenomic survey because of its sheer size and the novel functional diversity it revealed, especially in terms of the broader photosynthetic capability of oceanic microbes [3].

The genus *Burkholderia* are Gram negative bacteria which comprise over 30 species, many of which were formerly classified within the genus *Pseudomonas* [4]. *Burkholderia* are remarkably diverse and reside in a number of different niches including the soil and rhizosphere [4], river water [5], polluted terrestrial environments [6], endosymbiotic interactions with plants [4], opportunistic [7] and primary human infections [8]. Within the genus, the *Burkholderia cepacia* complex [9] form a closely related group of nine species that possess multiple ecologically beneficial properties such as the ability to degrade man-made pollutants (bioremediation), the protection of plants from pathogenic attack by nematodes and fungi (biological control), as well as the promotion of plant growth [4], [6], [7]. However, in contrast to their environmentally useful traits, *B. cepacia* complex bacteria are also particularly devastating pathogens in individuals with cystic fibrosis (CF) and also cause a variety of other opportunistic human infections [7].

Historically, our research has primarily focussed on examining the epidemiology and pathogenesis of *B. cepacia* complex bacteria in CF infection [7]. In particular, the ability of these pathogens to spread from one CF patient to another led to the early application of strain typing methods such as pulsed-field gel electrophoresis (PFGE) and PCR-based fingerprinting to track infection and develop infection control measures to prevent transmission [7], [10]. To further examine the global population biology and epidemiology of *B. cepacia* complex bacteria, we recently developed a multilocus sequence typing (MLST) scheme capable of both strain and species identification across the *B. cepacia* complex [11]. MLST is a very powerful strain typing technique based on nucleotide sequence analysis of several house-keeping genes from which unique allelic profiles known as sequence types (ST) are derived for genetically distinct strains [12]. Since MLST is based on nucleotide sequence and not DNA fragment pattern matching (like PFGE or PCR fingerprinting), the method is highly portable and STs can be compared using web-based databases that enable the global spread of bacterial pathogens to be tracked [12], [13]. In addition, sequence types may also be generated without the need to culture bacteria since PCR is used to amplify the MLST genes from a sample, an attribute which has made the method particularly useful when there is an urgent need to trace the causative agent of infection such as in bacterial meningitis [12].

Several *B. cepacia* complex genomes, including the *Burkholderia* SAR-1 metagenome, have been recently determined [7] and we had derived a sequence type for each from the available genomic sequence data. The identification of cultivable isolates that matched the hypothetical *Burkholderia* SAR-1 metagenome at all seven MLST loci occurred serendipitously as we examined *B. cepacia* complex strains in our collections. The availability of these cultivable strains provided us with the opportunity to examine their epidemiological background, taxonomy, genomic content, and ability to growth in seawater, all with a view to exploring the controversy of why *Burkholderia* SAR-1 had appeared in the Sargasso Sea dataset [2]. In addition, since the study of the *Burkholderia* genus is one of our primary interests, the matching cultivable strains also brought the opportunity to bring life to a hypothetical metagenome, which without viable surrogates was of limited practical use to our research community.

## RESULTS

### Identification of the matching cultivable isolates

The cultivable isolates found to match the *Burkholderia* SAR-1 metagenome are described in Table 1. Characterisation of *B. cepacia* complex bacteria was performed as part of ongoing collaborative international studies examining the epidemiology (in human infection) and environmental distribution of these microorganisms [7], [9]–[11], [14]–[18]. The MLST strain identification scheme examines nucleotide polymorphisms in seven genomically disparate housekeeping genes (ATP synthase β chain, *atpD*; glutamate synthase large subunit, *gltB*; DNA gyrase B, *gyrB*; Recombinase A, *recA*; GTP binding protein, *lepA*; Acetoacetyl-CoA reductase, *phaC* and Tryptophan synthase, *trpB*), and uses the resulting allelic profiles to assign a clonal sequence type (ST) to each unique strain [11]. After *in silico* analysis of the latter target genes within the *Burkholderia* SAR-1 metagenome, this uncultured hypothetical strain was designated as ST102. During initial analysis of cultivable strains, two isolates (LMG 23255 and LMG 23361; Table 1) were found to match the SAR-1 metagenome at all seven MLST loci, conforming to the same ST102 clone. Nine additional isolates, all deriving from human infections in North and South America, and Europe, were also identified as strain ST102 during subsequent typing. Interestingly, this analysis revealed that the ST102 clone was involved in a widespread outbreak of *Burkholderia* in the United States, ultimately traced to contaminated oxymetazoline nasal spray (represented by isolate AU7143; Table 1) [19]. In Spring 2004, the US Centers for Disease Control released a public notification of this outbreak and the contaminated product was recalled by its manufacturer [19]. Another ST102 isolate, LMG 23252 (Table 1), was part of an outbreak that was also linked to contamination by *Burkholderia* growth in reverse osmosis tubing of a water reservoir supplying a renal dialysis machine in São Paulo, Brazil [20].

**Table 1:** Cultivable *B. cepacia* complex ST102 strains matching the SAR-1 metagenome

*B. cepacia* complex strain	Source	Geographic location	Reference for original isolation of strain
LMG 23255 a	Cystic fibrosis	Czech Republic	This study
LMG 23361 a	Sheep mastitis	Spain	[34]
LMG 23250 a	Cystic fibrosis	Belgium	[35]
LMG 23251 a	Cystic fibrosis	Austria	This study
LMG 23252 a	Non-cystic fibrosis human infection	Brazil	Outbreak in a renal dialysis unit [20]
LMG 23253 a	Cystic fibrosis	Italy	[36]
AU2168	Cystic fibrosis	USA	This study
AU2176	Cystic fibrosis	USA	This study
AU2945	Cystic fibrosis	USA	This study
AU3443	Cystic fibrosis	USA	This study
AU7143	Non-cystic fibrosis human infection	USA	Contaminated nasal spray outbreak [19]

a All LMG strains are available from the Belgium Coordinated Collections of Microorganisms (BCCM; http://bccm.belspo.be/).

### Taxonomic identity of the cultivable isolates

The *B. cepacia* complex are a group of very closely related bacterial species that share significant phenotypic overlap and hence accurate identification of these bacteria requires a combination of both conventional biochemical assays and specific molecular tests [9]. Since 1997, their taxonomy has changed considerably with the complex currently comprising nine formally classified species [7]. Using the cultivable clones we sought to define the taxonomic status of the *Burkholderia* SAR-1 metagenome. The level of DNA-DNA hybridisation between *B. cepacia* complex bacteria is a fundamental component of their taxonomic differentiation, with strains possessing 70% or greater DNA-DNA reassociation values and phenotypic/genotypic commonality being classified as a species [9]. Payne et al. [16] placed the SAR-1 metagenome within *B. cepacia* complex Group K [18] by phylogenetic analysis of its *recA* sequence.

**Figure 1:**
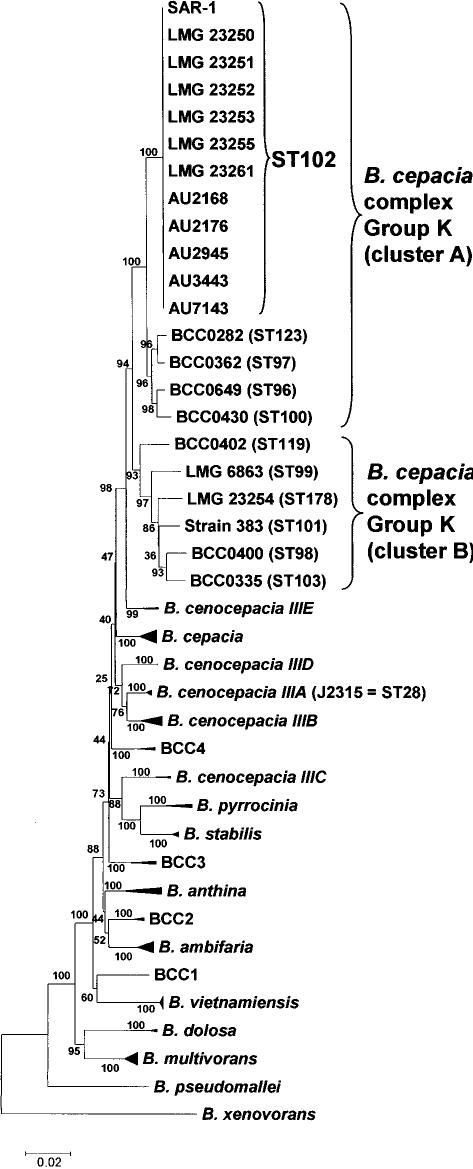
Phylogenetic analysis of SAR-1 and the cultivable ST102 isolates. Phylogenetic analysis of the concatenated MLST loci was performed on a collection of representative *B. cepacia* complex strains as described [11]. *B. cepacia* complex Group K, its separation into two clusters (A and B), and the position of the ST102 clones and SAR-1 metagenome. The positions of the genome reference strains, *B. cepacia* complex Group K strain 383 and *B. cenocepacia* J2315, are also indicated. Bootstrap values and genetic distance scale are shown.

MLST offers a significant advance over the identification methods described above in that it has proven capable of both strain differentiation as well as speciation of the currently classified *B. cepacia* complex taxonomic groups [11]. Analysis of the concatenated nucleotide sequence of the seven MLST loci demonstrated that the phylogenetic position of the SAR-1 metagenome and the cultivable ST102 clones resided within *B. cepacia* complex Group K (Fig. 1). This distinct phylogenetic group was originally described as closely related to *B. cepacia* (genomovar I) [18]. DNA-DNA hybridisation between the ST102 strains and other *Burkholderia* was examined to correlate the MLST-observed phylogeny with potential taxonomic differences. The ST102 strains LMG 23255 and LMG 23361 (Table 1) possessed a high DNA-DNA hybridisation value of 83% corroborating their clonality; comparison of these two strains with *B. cepacia* complex Group K strain 383 (a genome sequenced strain, see below) produced DNA-DNA hybridisation values of 61% and 62%, respectively. The phylogeny produced by MLST sequence data divides Group K strains into two distinct clusters, with the ST102 clones residing in cluster A and strain 383 grouping within cluster B (Fig. 1). Together with the DNA-DNA hybridisation values, these data suggest that Group K may constitute two novel *B. cepacia* complex species with the ST102 isolates and *Burkholderia* SAR-1 metagenome falling into *B. cepacia* complex Group K subgroup A; further detailed polyphasic taxonomic analysis [9] will be required to formalize this designation.

### Nucleotide sequence and genomic identity of the cultivable clones

**Figure 2:**
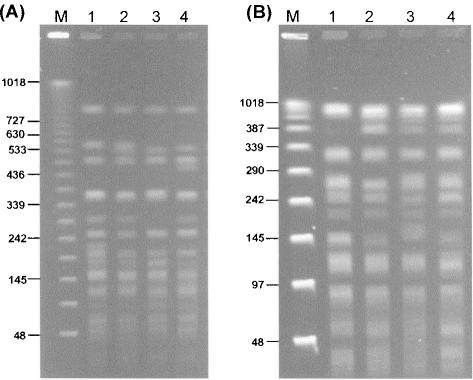
Macrorestriction analysis of the cultivable ST102 strains. The genomic macrorestriction fingerprint profiles resulting from restriction digestion with *Spe*I (Panel A) and *Xba*I (Panel B) are shown for the following strains in each lane: 1, LMG 23255, 2, LMG 23250, 3, LMG 23251, and 4, LMG 23253. Molecular size markers were run in the lane M and the size of relevant bands is indicated in kb.

The 100% nucleotide identity of all seven MLST loci indicated that the genomic sequence and content of the ST102 clones and *Burkholderia* SAR-1 metagenome should be essentially identical. To explore this assumption, the cultured strains were subjected to sequence analysis of random loci and also compared using several genomic mapping techniques. Nucleotide sequence analysis of random restriction fragments derived from ST102 isolates LMG 23255 and LMG 23361 (10 fragments from each strain) demonstrated 100% identity with the *Burkholderia* SAR-1 metagenome (see Supporting Table S1). The 100% match observed across a total of 3082 bases from random genomic regions corroborated the complete identity seen with the 2773 bases sampled from the house-keeping genes targeted by MLST. Genome macrorestriction followed by PFGE separation of DNA fragments was also used to examine the clonality of the ST102 in terms of genomic content and arrangement. The cultivable strains possessed genomic fingerprints that were considered identical (>70% genomic fingerprint similarity coefficients) when conventional strain typing criteria [10], [16], [21], [22] were applied to their analysis (Fig. 2). Minor alterations in the macrorestriction profiles of ST102 strains were present (Fig. 2); however, these were typical of genomic rearrangements (mediated by insertion sequences and other mobile entities) that have been observed to occur frequently during laboratory culture of *Burkholderia* species [7], [22]. Also in common with other *Burkholderia* genomes [7], strains LMG 23255 and LMG 23361 possessed a multireplicon organisation, comprising three chromosomes that were approximately 3.8, 3.1 and 1 Mb in size (data not shown). This total genome size is close to the 8.45 Mb *Burkholderia* SAR-1 metagenome and within the range of 6 to 9 Mb observed in other *B. cepacia* complex bacteria [7].

### Comparison of the *Burkholderia* SAR-1 metagenome to related *Burkholderia* genomes

The degree of completion and quality of the *Burkholderia* SAR-1 metagenome was not known. The availability of several closely related *B. cepacia* complex species genomes [7] enabled their comparison to the metagenome to assess its quality and utility as a tractable genomic resource. The genome sequence of strain 383, originally isolated from forest soil in Trinidad [23], represents the closest sequenced relative to *Burkholderia* SAR-1, since it is also a member of *B. cepacia* complex Group K (see Fig. 1). The genomes of *Burkholderia* SAR-1 and strain 383 were compared *in silico* to *Burkholderia cenocepacia* strain J2315 (representing a manually annotated and entirely finished reference genome) in order to estimate the gene content and homology of each strain. After alignment of the genomes, the presence of homologs of each J2315 gene in either the strain 383 or SAR-1 sequences was scored; if there was no corresponding J2315 homolog in these *B. cepacia* Group K sequences, the gene was designated as absent (see Methods and Materials; Table 2). The absence of genes homologous to J2315 in the 383 and SAR-1 sequences did not preclude the presence of novel DNA insertions in each of latter strains at given genomic positions. However, such unique DNA was not counted in our analysis as we were aiming to define the extent to which the *B. cepacia* SAR-1 metagenome overlapped other *Burkholderia* genomes in terms of the known gene content of a reference strain.

**Table 2:** Gene content of *Burkholderia* SAR-1 and *B. cepacia* 383 in comparison to the reference genome of *B. cenocepacia* J2315

*B. cenocepacia* J2315 genes encoded on (number of genes):	Number of homologous genes (%) in:			
	*B. cepacia* 383		*Burkholderia* SAR-1	
	Present	Absent	Present	Absent
Chromosome 1 (3463 genes)	2928 (85%)	535 (15%)	2832 (82%)	631 (18%)
Chromosome 2 (2860 genes)	2188 (77%)	672 (23%)	2139 (75%)	721 (25%)
Chromosome 3 (771 genes)	313 (41%)	458 (59%)	391 (51%)	380 (49%)
Total (7094 genes)	5429 (77%)	1665 (23%)	5362 (76%)	1732 (24%)

**Figure 3:**
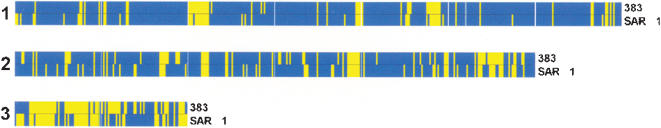
Genomic distribution of *Burkholderia* SAR-1 and *B. cepacia* 383 genes homologous to *B. cenocepacia* J2315. The distribution of homologous genes (blue) and absent genes (yellow) are shown for each *B. cepacia* complex Group K genome (indicated on the right). The panels correspond to the three J2315 chromosomes (chromosome number indicated on the left).

The SAR-1 and 383 genomes shared notable synteny, with orthologous genes encoded at the same relative position on each chromosome when compared with *B. cenocepacia* J2315 (Fig. 3; Supporting data file Genome comparison J2315×SAR×383.xls). Both strains also shared putative deletions or insertions at conserved sites leading to mosaic genome structures which have also been observed in *Pseudomonas aeruginosa* [21], [24]. Chromosome 1 possessed the greatest level of homology for both strains, with chromosome 3 having the least homology and being largely made up of DNA unique to each Group K strain and not shared with *B. cenocepacia* (Fig. 3, Table 2). Analysis of the *Burkholderia pseudomallei* genome demonstrated that the largest chromosome of this *Burkholderia* species also encoded the majority of conserved housekeeping genes [8] and this phenomenon also appears to be mirrored by the J2315, SAR-1 and strain 383 genomes where the greatest degree of gene conservation is seen on chromosome 1 (Fig. 3, Table 2). The bioinformatic analysis demonstrated that the metagenomic sequence of *Burkholderia* SAR-1 was essentially complete sharing 98.7% (5362/5429; Table 2) of conserved genes present in J2315 with *B. cepacia* complex Group K strain 383.

### Analysis of *Burkholderia* growth in conditions reflective of sea water

Using the cultivable SAR-1 *B. cepacia* complex Group K matches and other *Burkholderia* species, the controversy of whether these bacteria can grow and survive in the open ocean [2] was also examined. Growth experiments in minimal medium (basal salts medium; BSM) with salt concentrations typical of sea water (an average of 3.5% NaCl) showed that while all *Burkholderia* species can replicate at this salt level, only *Burkholderia gladioli* grew reasonably well (Table 3). However, all *Burkholderia* tested grew very poorly in actual sea water including the sole saltwater marine-associated isolate we have in our collection, strain LMG 23254 (Table 3), which is unrelated to SAR-1 (Fig. 1). At salt concentrations above the sea water average, 77% of the *Burkholderia* species tested grew to less than 10% of the control cultures without additional salt. In contrast, *P. aeruginosa* was not effected by 3.5% NaCl or concentrations up to 5% (Table 3), corroborating emerging evidence that *P. aeruginosa*, although historically thought of as a terrestrial or freshwater organism, may be considered a natural inhabitant of coastal marine environments [25]. Also when the salt concentration was increased to sea water level (3.5%), the average doubling time in a rich medium of three *B. cepacia* complex Group K strains increased by greater than 5 fold (Supporting Table S2); this very slow replication rate suggests they would be highly uncompetitive in nutrient-limited sea water conditions. In contrast, the growth of *P. aeruginosa* was relatively unaffected by increasing salt concentrations with only a 1.3 fold increase in doubling time at 3.5% salt (Supporting Table S2). Survival experiments demonstrated that the cultivable ST102 clones remained viable at 3.5% salt for up to 26 days but lost viability beyond this time point. In contrast, *P. aeruginosa* remained viable up to the last sampling point of 41 days (data not shown).

**Table 3:** Salt tolerance of *Burkholderia* species

Species	Strain	Generation time (minutes)		Percentage of growth yield a		
		BSM	3.5% NaCl in BSM	3.5% NaCl in BSM	5% NaCl in BSM	sea water
*B. cepacia* complex						
*B. cepacia* complex Group K	LMG 23255 (ST102)	601	2674	17.4	5.7	21.3
*B. cepacia* complex Group K	LMG 23361 (ST102)	574	4721	11.9	3.9	11.1
*B. cepacia* complex Group K	LMG 23254	791	1115	21.9	4.3	8.8
*Burkholderia cepacia*	LMG 1222 ^T^	431	1442	13.0	4.2	4.6
*Burkholderia multivorans*	LMG 13010 ^T^	430	1383	27.1	21.2	20.7
*Burkholderia cenocepacia*	LMG 18863	588	1928	27.1	13.7	19.8
*Burkholderia stabilis*	LMG 14294 ^T^	926	3972	31.8	9.6	15.7
*Burkholderia vietnamiensis*	LMG 16232^T^	687	1916	18.7	12.1	12.3
*Burkholderia dolosa*	LMG 18943^T^	572	1599	19.8	9.3	18.3
*Burkholderia ambifaria*	LMG 19182^T^	534	1592	29.7	5.6	9.1
*Burkholderia anthina*	LMG 20980^T^	375	1743	21.7	7.3	8.0
*Burkholderia pyrrocinia*	LMG 14191^T^	530	1228	18.4	8.1	8.0
*Burkholderia* species						
*Burkholderia fungorum*	LMG 16225 ^T^	549	3327	11.1	6.9	31.6
*Burkholderia graminis*	LMG 18924 ^T^	687	2023	13.4	8.7	16.7
*Burkholderia gladioli*	LMG 2216 ^T^	411	1002	63.5	21.2	15.2
*Burkholderia glathei*	LMG 14190 ^T^	410	5618	8.0	9.8	20.6
*Burkholderia thailandensis*	LMG 20219 ^T^	507	5624	8.9	3.6	11.5
*Burkholderia plantarii*	LMG 9035 ^T^	700	3464	19.4	8.6	23.0
Control species						
*Pseudomonas aeruginosa*	PAO1	234	846	109	68.7	33.0

a Percentage of growth yield observed by optical density calculated by comparison to a control culture grown in standard BSM medium for 48 hours.

## DISCUSSION

Using MLST we identified eleven, widely distributed, cultivable bacterial isolates belonging to the *Burkholderia cepacia* complex [7] that were clonal matches to the uncultured *Burkholderia* SAR-1 metagenome [3]. We have demonstrated that MLST, as a nucleotide sequenced-based strain typing method, has the power to bring life to a microbial metagenome by the identification of matching cultivable clones, which may now be used as viable surrogates for research using the metagenome. The phenotype and biological heritage of the cultivable isolates also add weight to the argument that the presence of *Burkholderia* SAR-1 within the Sargasso Sea metagenomic [3] dataset most likely represents the results of sample contamination [2].

The matching cultivable isolates, from North and South America, and Europe, were isolated from sheep mastitis and human infections (including patients with cystic fibrosis) and included strains linked to contamination of nasal sprays [19] and dialysis equipment [20] (Table 1). *B. cepacia* complex bacteria have a propensity towards growth as a contaminant and have historically been isolated from a wide range of commercial sources including disinfectants [26] and cosmetics [27]. In addition, these bacteria are also capable of degrading many organic pollutants and are frequently isolated from contaminated industrial sites and fuel tanks [6]. Interestingly, the company that manufactured the *Burkholderia* contaminated nasal spray (Table 1) was based in Miami, USA [19], leading to further speculation that effluent of ships servicing this industrial site could have been in the vicinity of the Sargasso Sea, specifically polluting the sample site from which *Burkholderia* SAR-1 was derived. Given the ability of the ST102 strain to contaminate water lines [20] it is also conceivable that the metagenome derived from contaminated water onboard the yacht used for sampling [3]. All these observations contribute to the notion that *Burkholderia* SAR-1 was probably a sample-specific contaminant or resulted from shipboard contamination during analysis [2].

The ST102 clones were only capable of very slow growth and limited survival at salt concentrations typical of sea water; other *Burkholderia* species also failed to grow well in these conditions (Table 3). Although we have characterised several thousand *B. cepacia* complex strains over the past 15 years [7], [9]-[11], [14]-[18], only one of these, *B. cepacia* complex Group K strain LMG 23254 (Fig. 1; Table 3), was apparently derived from a salt-water marine environment. Strain LMG 23254 was isolated from an enrichment of sea water in media containing polyhydroxybutyrate (PHB) as part of a study examining PHB degrading enzymes [28]; its was not recovered as part of systematic study of oceanic microbial diversity. There is no description of the exact source of the sea water (other than the Sea of Japan) in the original study [28], hence it could have easily represented the remnants of terrestrial run-off. Venter et al. [3] cite a study examining fatal *B. pseudomallei* (the causative agent of human meliodosis) infections in marine mammals as a plausible mechanism of *Burkholderia* transfer to the open ocean. However, the study in question by Hicks et al. [29], was a survey of captive marine mammals within oceanariums in Hong Kong; the author's concluded that the incidence of *B. pseudomallei* infection was greatest during periods of heavy rain and its source was probably soil sediments (where *B. pseudomallei* resides; [8]) that washed off into the holding pools. There is currently no documented evidence to indicate that the highly infectious *B. pseudomallei* or other *Burkholderia* species naturally infect or colonise marine mammals in the wild. The lack of marine strains in our collection and the absence of its description as an oceanic species within the extensive studies of this niche [2], suggest that it is not normally part of the microbial community which live in the sea.

“Breathing life” is a phrase that has often been used as a fund raising slogan by cystic fibrosis charities, many of which have supported research on *B. cepacia* complex bacteria as highly problematic pathogens in this disease. The ability of MLST to work from a starting point of nucleotide sequence and trace back to cultivable bacteria which are absolute genetic matches this DNA, is testament to its resolving power as a typing tool. Our original finding could not have been achieved with conventional strain typing methods based on DNA fragment pattern matching. To date, MLST type approaches have had limited application to questions of population biology in environmental microbiology, where the majority of studies use phylogenetic analysis based on single genes such as the 16S rRNA gene [1] or *recA* [16] as a means to examine diversity. Since polymorphism within these genes alone are unlikely to discriminate between strains, most studies inherently work at the species level of discrimination and shed little light on what the actual population biology at the strain level is within a given environment. Depending on the quality of assembled DNA, metagenomics offers a level of discrimination that can potentially differentiate strains within a species. Protein coding anchor genes such as *recA* and *gyrB* are often examined to resolve sample diversity in metagenomic studies [1]. If two or more such genes are encoded on a single metagenomic assembly, then they can begin to contribute towards assigning sequence type to the originating microorganism. However, to enable further matching of cultivable isolates to metagenomic sequences, the environmental microbiology research community will need to begin to establish greater sequence datasets for MLST target genes within cultivable isolates that reside in culture collections.

Irrespective of the fact that *Burkholderia* SAR-1 was probably not part of the natural microbial populations in the Sargasso Sea, the metagenomic study by Venter et al. [3] has had considerable impact on the field of oceanic microbiology by defining millions of unknown genes and hundreds of previously unknown bacterial species. It has also had an impact on the *Burkholderia* research community by providing an additional genome that we have now shown is essentially complete. The identification of cultivable clones which match this metagenome and their deposition in a public culture collection brings further utility to the *Burkholderia* SAR-1 metagenomic nucleotide sequence as a tractable genetic resource.

## MATERIALS AND METHODS

### Bacterial strains

The *B. cepacia* complex strains examined in this study were drawn from the following collections: Cardiff University, Cardiff, Wales, UK [7], [11], [15], [16], [30]; the European *B. cepacia* complex Referral Laboratory, Gent, Belgium [9], [17], [18] and US *B. cepacia* Research Laboratory and Repository, Ann Arbor, Michigan, USA [14]. Bacteria were cultured and identified using polyphasic taxonomic approaches exactly as described previously [9], [11], [15]. The cultivable ST102 isolates matching the Sargasso Sea metagenome are described in Table 1. Additional strains analysed from *B. cepacia* complex Group K were: LMG 23254 (ST178), an isolate enriched from seawater in the Sea of Japan [28]; cystic fibrosis strains, BCC0649 (ST96), BCC0362 (ST97), BCC0340 (ST100), BCC0335 (ST103), and BCC0282 (ST123); and environmental strains BCC0400 (ST98), BCC0402 (ST119) and LMG 6863 (ATCC 17460; ST 99). Additional *Burkholderia* species reference strains used in the salt-growth experiments are described in Table 3 and Supporting Table S2 *. P. aeruginosa* PAO1 was used as a control species. All LMG strains are available from the Belgium Coordinated Collections of Microorganisms (BCCM; http://bccm.belspo.be/).

### Multilocus sequence typing, phylogenetic analysis and DNA sequencing

Amplification and sequence analysis of the seven MLST gene loci (*atpD*; *gltB*; *gyrB*; *recA*; *lepA*; *phaC* and *trpB*) were performed exactly as described [11]. Nucleotide sequences of each allele, allelic profiles and sequence types for all the strains analysed in this study are available from http://pubmlst.org/bcc/. Phylogenetic analysis using the program MEGA Version 3 http://www.megasoftware.net/) was performed as described [11]. Small insert (100-500 bp) libraries of LMG 23255 and LMG 23361 were prepared in plasmid pUC18 after digestion of chromosomal DNA using *Sau3*A1 and sequenced as described [31]. Sequences were compared to the Sargasso Sea data set and GenBank using the National Centre for Biotechnology Information Basic Local Alignment Search Tools (BLAST).

### Genomic fingerprinting and analysis

Macrorestriction and separation of the DNA fragments by PFGE were performed following the procedures outlined in previous studies [16], [17], [21], [30]. Estimation of genome size and visualisation of the chromosomal replicons was carried out as described [15].

### Bioinformatic analysis and genome comparison

The metagenome of *Burkholderia* SAR-1 was obtained from GenBank (accession number NS_000028). The genome of *B. cepacia* complex Group K strain 383 [23] (ATCC 17760) was produced by the US Department of Energy Joint Genome Institute (http://www.jgi.doe.gov/) and is available from the microbial genomes web page http://genome.jgi-psf.org/finished_microbes/bur94/bur94.home.html. *B. cenocepacia* J2315 was used as a reference genome because unlike strain 383, it is completely finished and is the only genome from the *B. cepacia* complex to have undergone a detailed manual annotation. The J2315 sequence data was produced by the Pathogen Sequencing Group at the Sanger Institute and can be obtained from http://www.sanger.ac.uk/Projects/B_cenocepacia/. Comparison of the SAR-1 and 383 genomes was performed using the Artemis Comparison Tool Version 4 (ACT) [32] and TBLASTX comparison files generated as described in the ACT user manual (http://www.sanger.ac.uk/Software/ACT/v4/manual/). Genes which demonstrated greater than 70% identity over more than half of their coding length to the corresponding *B. cenocepacia* J2315 gene were marked as homologous and present in strain 383 or SAR-1. Multiple copy genes such those on IS elements or rRNA genes were counted only once. Gene presence and absence data was recorded in a spreadsheet and the genome comparison graphic (Fig. 3) generated by colouring the present genes blue and absent genes yellow, and copying the spreadsheet columns as a bitmap [24]. The spreadsheet marking the orthologs shared by the metagenome, strain 383 and *B. cenocepacia* J2315 is available as a supporting data file (Dataset S1).

### Growth in high salt concentrations and seawater

Sodium chloride (from 0.5% to 5%) was added to either Tryptic Soya Broth (TSB) or a minimal basal salts medium [33] (BSM) containing 14.3 mM glucose and 0.05% casamino acids. Fresh, filter-sterilised (0.22 µM exclusion) sea water was obtained from Whitesands Bay, St. Davids, Pembrokeshire, Wales, UK (coordinates N51:53:26 and W5:18:27). Growth experiments were performed in 150 µl of media contained within a 96-well flat bottomed polystyrene plate. Overnight starter cultures were standardised to an optical density of 0.5 at 600 nm (approximately 10^8^ colony forming units per ml) and 10 µl used to inoculate each well. Replicate cultures (three) were incubated in an automatic plate reader (MRX Revelation, Dynex Technologies Ltd) at 30°C and grown with shaking for 5 seconds every 10 minutes; optical density readings (630 nm) were taken every 10 minutes to monitor growth for up to 72 hours. Temperatures reflective of the Sargasso Sea (18 to 25°C) [3] were evaluated, however, the *Burkholderia* growth rates observed were too slow and inconsistent to obtain statistically valid results. The culture doubling time was calculated during the maximal growth phase occurring between 7 and 11 hours for each respective salt concentration. Survival in the presence of salt (3% and 5%) was evaluated by inoculation of 100 µl of a standardised starter cultures into 15 ml tubes containing 5 ml of BSM and incubating the cultures with shaking (150 rpm) at 25°C. Surviving bacteria were plated for growth on Tryptic Soya Agar at regular intervals up to 41 days.

## Supporting Information

Table S1 Random loci from ST102 strains matching Burkholderia SAR-1
(0.06 MB DOC)

Table S2 Effect of salt concentration on the doubling time of Burkholderia strains and P. aeruginosa
(0.05 MB DOC)

Dataset S1 Genome comparison J2315 x 383 x SAR-1. This is an Microsoft Excel spreadsheet marking the orthologs shared by the SAR-1 metagenome, strain 383 and *B. cenocepacia* J2315.
(9.71 MB XLS)
