# Supplementary material for: Multilocus Sequence Typing Breathes Life into a Microbial Metagenome
Source: PLoS One. 2006 Dec 20;1(1):e17. doi: 10.1371/journal.pone.0000017 (PMC1762331; doi:10.1371/journal.pone.0000017)
Supplement: Table S1 — Random loci from ST102 strains matching Burkholderia SAR-1 (0.06 MB DOC) [file pone.0000017.s001.doc]

**Table S1. Random loci from ST102 strains matching *Burkholderia*** SAR-1

| **Strain and clone number:** | **Fragment length (bp)** | **Putative function of homologous gene in**  ***Burkholderia* *cepacia* Group K strain 383 genome:** | **GenBank Protein ID:** |
| --- | --- | --- | --- |
| **LMG 23255** | | | |
| 14_a | 154 | Phosphoglycerate mutase | ABB13149.1 |
| 34_a | 157 | Hypothetical protein | ABB12090.1 |
| 34_b | 217 | Hypothetical protein | ABB12389.1 |
| 104_a | 199 | Penicillin-binding protein 1A | ABB10330.1 |
| 114_a | 90 | ATPase | ABB09990.1 |
| 134_a | 82 | Hydrophobe efflux pump | ABB10412.1 |
| 134_b | 120 | 4-hydroxyphenylpyruvate dioxygenase | ABB11739.1 |
| 154_a | 130 | Conserved hypothetical protein | ABB12855.1 |
| 234_a | 278 | Fimbrial protein | ABB08369.1 |
| 274_a | 273 | ABC amino acid transporter | ABB07356.1 |
| **LMG 23361** |  |  |  |
| 16_c | 80 | ABC transporter | ABB08592.1 |
| 66_a | 219 | Pilus assembly protein | ABB08259.1 |
| 106_b | 88 | Serine protein kinase | ABB08346.1 |
| 106_c | 106 | GCN5-related N-acetyltransferase | ABB11231.1 |
| 116_b | 77 | TonB-dependent haemoglobin receptor | ABB10337.1 |
| 136_a | 260 | Transcriptional regulator | ABB11479.1 |
| 146_a | 82 | ABC branched chain amino acid transporter | ABB12063.1 |
| 146_c | 136 | MviN-like virulence factor | ABB09476.1 |
| 246_a | 67 | Hypothetical protein | ABB06465.1 |
| 296_a | 267 | Transcriptional regulator | ABB12144.1 |
