# Supplementary material for: Multilocus Sequence Typing Breathes Life into a Microbial Metagenome
Source: PLoS One. 2006 Dec 20;1(1):e17. doi: 10.1371/journal.pone.0000017 (PMC1762331; doi:10.1371/journal.pone.0000017)
Supplement: Table S2 — Effect of salt concentration on the doubling time of Burkholderia strains and P. aeruginosa (0.05 MB DOC) [file pone.0000017.s002.doc]

**Table S2. Effect of salt concentration on the doubling time of *Burkholderia* strains and *P. aeruginosa***

| **Strain** | **Mean doubling time (minutes) in Trytic Soya Broth containing the**  **following salt concentrations (± standard deviation)** | | | | | | |
| --- | --- | --- | --- | --- | --- | --- | --- |
|  | **0.5%** | **2%** | **3%** | **3.5%** | **4.0%** | **4.5%** | **5%** |
| ST102 isolate  LMG 23255 | 221 (13) | 427 (7) | 688 (157) | 1186 (358) | 1583 (124) | 1664 (123) | 2740 (429) |
| ST102 isolate  LMG 23361 | 226 (8) | 530 (119) | 940 (180) | 2093 (61) | 4103 (567) | 5693 (527) | 14794 (3704) |
| LMG 23254  (Marine isolate) | 581 (62) | 1040 (144) | 1204 (123) | 1780 (170) | 2302 (463) | 4050 (1278) | 8819 (3198) |
| *P. aeruginosa*  PAO1 | 274 (20) | 311 (20) | 348 (54) | 367 (54) | 358 (4) | 388 (21) | 449 (24) |
